# Artificial Intelligence (AI) Competency and Educational Needs: Results of an AI Survey of Members of the European Society of Pediatric Endoscopic Surgeons (ESPES)

**DOI:** 10.3390/children12010006

**Published:** 2024-12-24

**Authors:** Holger Till, Hesham Elsayed, Maria Escolino, Ciro Esposito, Sameh Shehata, Georg Singer

**Affiliations:** 1 Department of Pediatric and Adolescent Surgery, Medical University of Graz, 8036 Graz, Austria; hesham.elsayed@medunigraz.at (H.E.); georg.singer@medunigraz.at (G.S.); 2 Division of Pediatric Surgery, Federico II University Hospital, 80131 Naples, Italy; maria.escolino@unina.it (M.E.); ciroespo@unina.it (C.E.); 3 Department of Pediatric Surgery, University of Alexandria, Alexandria 5424041, Egypt; sameh_shehata@alexmed.edu.eg

**Keywords:** artificial intelligence, machine learning, image analysis, surgical planning, data bias, pediatric surgery, endosurgery

## Abstract

**Background**: Advancements in artificial intelligence (AI) and machine learning (ML) are set to revolutionize healthcare, particularly in fields like endoscopic surgery that heavily rely on digital imaging. However, to effectively integrate these technologies and drive future innovations, pediatric surgeons need specialized AI/ML skills. This survey evaluated the current level of readiness and educational needs regarding AI/ML among members of the European Society of Pediatric Endoscopic Surgeons (ESPES). **Methods**: A structured survey was distributed via LimeSurvey to ESPES members via email before and during the 2024 Annual Conference. Responses were collected over four weeks with voluntary, anonymous participation. Quantitative data were analyzed using descriptive statistics. **Results**: A total of 125 responses were received. Two-thirds (65%) of respondents rated their AI/ML understanding as basic, with only 6% reporting advanced knowledge. Most respondents (86%) had no formal AI/ML training. Some respondents (31%) used AI/ML tools in their practice, mainly for diagnostic imaging, surgical planning, and predictive analytics; 42% of the respondents used these tools weekly. The majority (95%) expressed interest in further AI/ML training, preferring online courses, workshops, and hands-on sessions. Concerns about AI/ML in pediatric surgery were high (85%), especially regarding data bias (98%). Half of respondents (51%) expect AI/ML to play a significant role in advancing robotic surgery, oncology, and minimally invasive techniques. A strong majority (84%) felt that the ESPES should lead AI education in pediatric surgery. **Conclusions**: This survey presents the ESPES with a unique opportunity to develop a competency map of its membership’s AI/ML skills and develop targeted educational programs, thus positioning the society to take the lead in AI education and the advancement of AI solutions in pediatric endosurgery.

## 1. Introduction

Artificial intelligence (AI) has shown transformative potential across various medical specialties, where it can significantly improve diagnostic precision, support surgical decision-making, and enhance overall patient outcomes [1]. There is a wide range of possible applications of AI in medicine, including machine learning for predictive analytics and decision support. For instance, ML has outperformed logistic regression for the prediction of surgical site infections [2]. The enhancement of diagnostic precision for appendicitis by ML models has been demonstrated in a variety of publications [3,4]. Computer vision is a field of AI focusing on enabling computers to interpret, understand, and process visual information, allowing machines to make sense of images and videos [5]. This method can specify regions of interest during laparoscopy, thereby assisting surgeons during minimally invasive surgery (MIS). This has already been demonstrated for common procedures such as laparoscopic cholecystectomy [6].

In particular, AI’s ability to assist in MIS through advanced image recognition, predictive analytics, and real-time decision support could make it an invaluable tool in the future. However, the successful adoption of AI mainly depends on the preparedness and competencies of the surgical community [1,7].

The European Society of Pediatric Endoscopic Surgery (ESPES), an international organization dedicated to advancing pediatric MIS, is ideally positioned to assess and promote the integration of AI into clinical practice. Understanding the current level of AI knowledge and usage among pediatric surgeons is crucial to ensure effective AI implementation in pediatric surgery. This survey aimed to evaluate the readiness of ESPES members by assessing their current AI knowledge, use of AI/ML in clinical practice, educational needs, and the barriers they face in adopting AI technologies. The ultimate goal is to identify areas for further education and future training by the ESPES to facilitate the integration of AI/ML solutions into pediatric endosurgery safely.

## 2. Materials and Methods

We developed a structured survey focusing on artificial intelligence (AI), especially image-based machine learning (ML) models that play an important role in endoscopic surgery. We defined six key categories: (1) demographics and background information, (2) general awareness and knowledge of AI/ML, (3) current use of AI/ML in clinical practice, (4) educational needs and training, (5) attitudes toward AI/ML, challenges, and barriers, and (6) future perspectives.

The survey featured a combination of multiple-choice and open-ended questions. Several adjustments were made for clarity and relevance after pilot testing the survey with a group of local pediatric surgeons. The final questionnaire was distributed via LimeSurvey.

Data Collection: The survey was disseminated via email to all ESPES members, promoted during the 2024 annual ESPES conference, and followed by a reminder email one week prior to survey closure. Responses were collected over a four-week period. Participation was voluntary, and all responses were anonymized to ensure confidentiality.

Data Analysis: Quantitative data were analyzed using descriptive statistics, including response rates and frequency distributions. Open-ended responses were subjected to thematic analysis to identify recurring themes regarding attitudes, barriers, and expectations related to AI adoption. Categorical data comparisons for different subgroups were analyzed with the chi-squared test. A *p*-value < 0.05 was considered statistically significant.

## 3. Results

### 3.1. Demographics and Background Information

A total of 307 ESPES members were invited. Out of these, 144 participated in the survey; 19 respondents did not answer any AI-related questions, leaving 125 responses included in the analysis. A total of 72 (58%) respondents were male and 53 (42%) were female; 13 (10%) respondents were younger than 30 years, 43 (35%) were aged between 31 and 40 years, 34 (27%) were between 41 and 50 years, and 35 (28%) were older than 50 years.

A total of 25 (20%) respondents stated that they have less than five years of experience in pediatric surgery, 22 (18%) have between 5 and 10 years, 37 (29%) between 11 and 20 years, and 41 (33%) more than 20 years. The vast majority of responders primarily work in an academic medical center (n = 98, 78%); the remaining 27 (22%) work in community hospitals or private practice. More than three quarters (n = 103; 82%) declared that they work in Europe, the remaining 22 (18%) work in Asia (n = 10, 8%), Africa (n = 8, 7%), North America (n = 3, 2%), and South America (n = 1, 1%).

### 3.2. General Awareness and Knowledge of AI/ML

**Figure 1:**
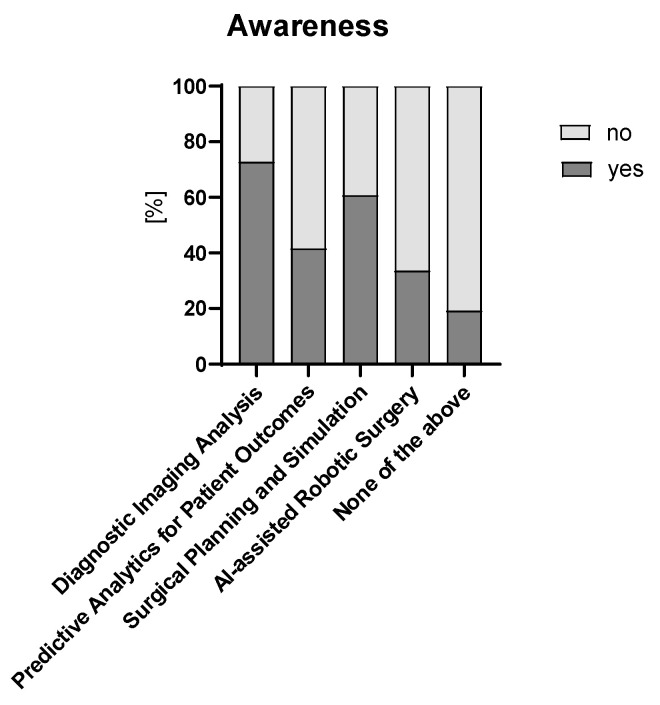
AI/ML applications participants are aware of in the context of pediatric surgery. All 125 participants answered this question.

Almost two thirds of respondents rated their general understanding of AI/ML as basic (n = 81, 65%), n = 9 (7%) as none, n = 28 (22%) as intermediate, and only n = 7 (6%) as advanced. Neither age group, years of experience, nor primary institution seemed to influence the general understanding of AI/ML (Supplementary Figure S1). Figure 1 depicts AI/ML applications participants are aware of in the context of pediatric surgery.

The vast majority stated that they did not receive any formal training in AI/ML (n = 107, 86%); n = 15 (12%) received some sort of training (n = 9 online courses, n = 9 workshops, n = 9 self-studies, and n = 8 formal academic studies). Three respondents (2%) did not answer the question.

### 3.3. Current Use of AI/ML in Clinical Practice

**Figure 2:**
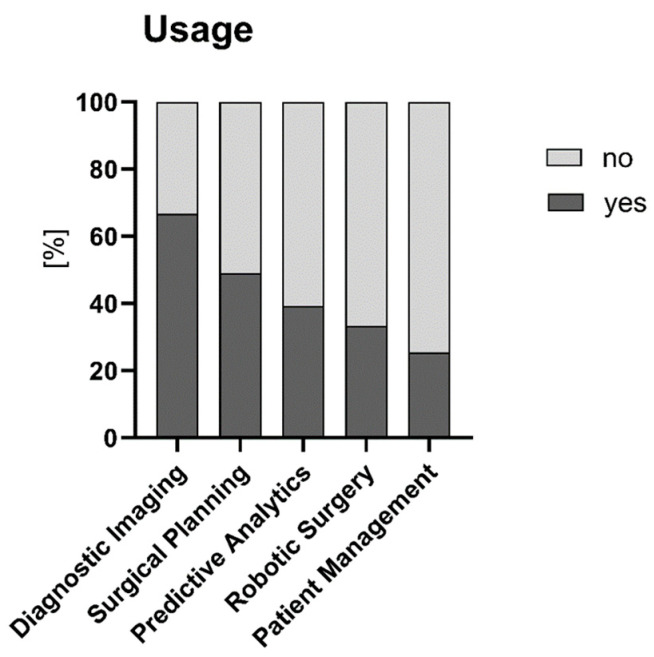
Areas in which AI/ML tools are used.

A total of 120 respondents answered the question whether they currently use AI/ML tools in their practice; over half of them (n = 65, 54%) stated they do not use any AI/ML tools, n = 37 (31%) use AI/ML tools, and n = 18 (15%) were not sure. Areas in which respondents use AI/ML applications are depicted in Figure 2.

### 3.4. Educational Needs and Training

**Figure 3:**
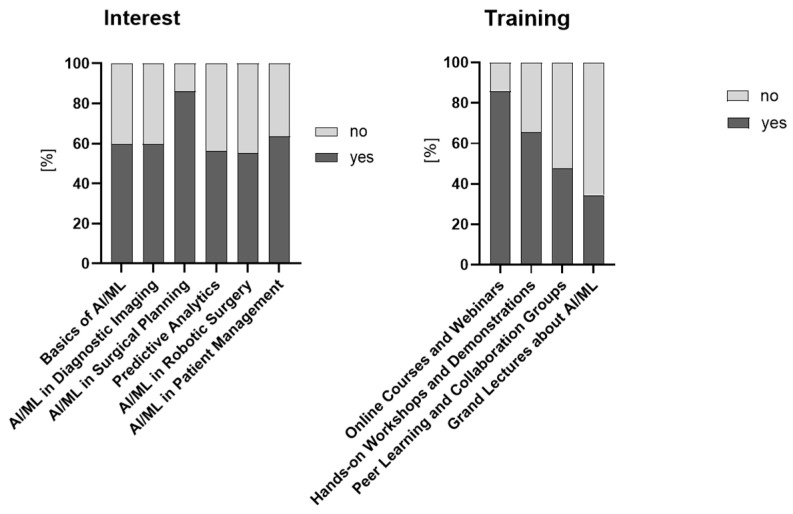
Specific areas and types of training respondents are interested in.

Of the 107 respondents who answered the question whether they would be interested in receiving more training in AI/ML, 102 (95%) answered yes. Specific areas of interest for further training and types of preferred training are depicted in Figure 3.

### 3.5. Attitudes Towards AI/ML, Challenges, and Barriers

**Figure 4:**
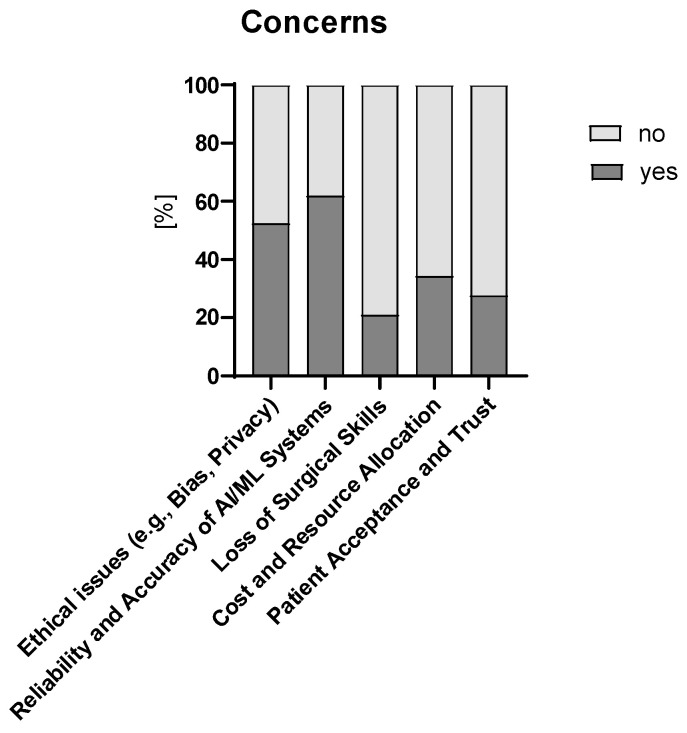
Specific concerns regarding AI/ML.

**Figure 5:**
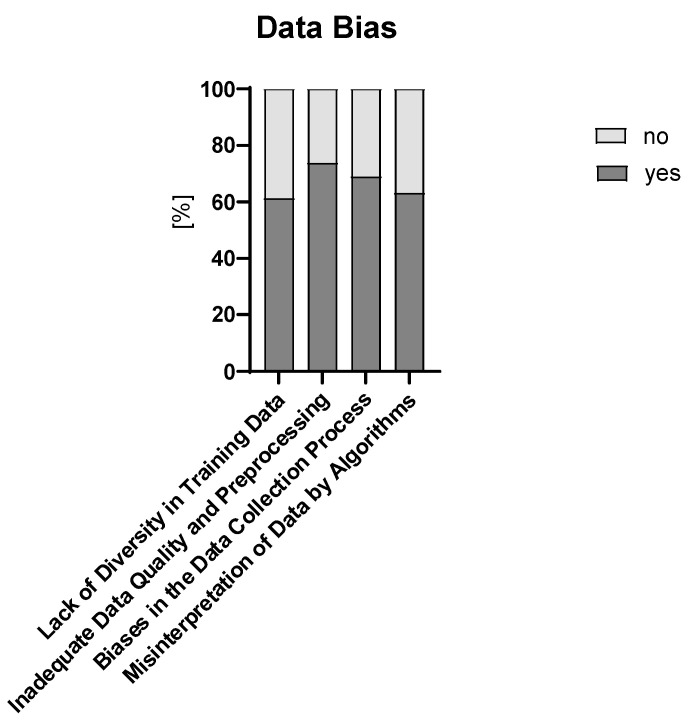
Primary causes of data bias in AI/ML algorithms used in healthcare.

More than two-thirds of participants stated that they have no or some concerns about implementing AI/ML technologies in their practice (no concerns, n = 38, 36%; some concerns, n = 52, 49%). The remaining respondents stated significant concerns (n = 9, 8%) or indifference (n = 7, 7%). Age group did not influence concerns towards the implementation of AI/ML (Supplementary Figure S2). These concerns mainly regarded ethical issues and the reliability and accuracy of AI/ML systems (Figure 4). The question about data bias revealed few respondents with no concerns (n = 2, 2%), 47% (n = 49) with some concerns, and 51% (n = 52) with significant concerns. Figure 5 shows the primary causes of data bias in AI/ML algorithms used in healthcare.

### 3.6. Future Perspectives

**Figure 6:**
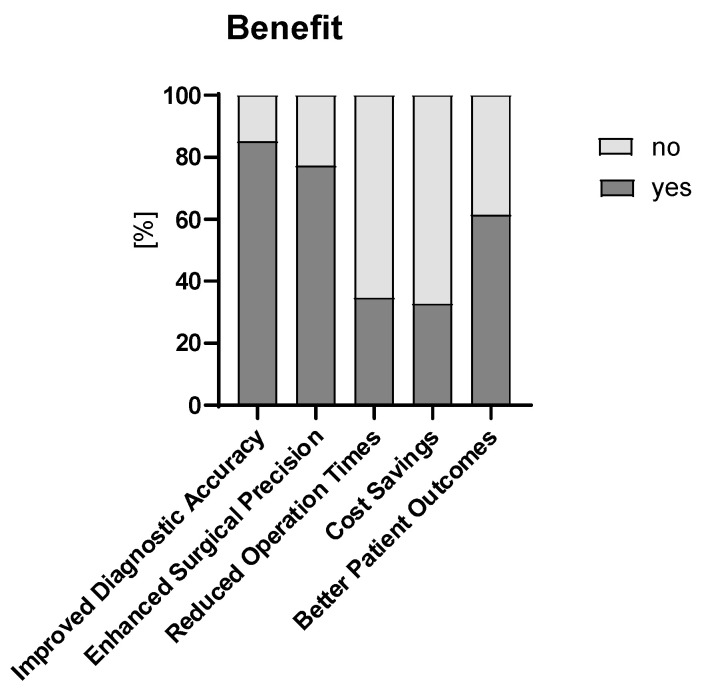
Primary benefits of integrating AI/ML into pediatric surgery.

Half of the respondents see an integral role of AI/ML evolving in pediatric surgery over the next 10 years (n = 52, 51%). A limited role was stated by n = 41 (41%), and a minimal role by n = 5 (5%). Three respondents (3%) were not sure. Opinions concerning the primary benefits of integrating AI/ML into pediatric surgery are depicted in Figure 6. In the open ended question “*Which endosurgery procedures and future advancements in AI/ML would you like to see implemented in pediatric surgery?*”, robotic surgery was listed 25 times, oncology 19 times, and minimal invasive surgery and endoscopy 8 times each. The vast majority of respondents expect the ESPES to take a leading role in education regarding AI in pediatric surgery (yes, n = 84, 84%; no, n = 4, 4%; not sure, n = 12, 12%).

## 4. Discussion

The most important findings of our survey were that while there is a certain use of AI/ML models, most EPSES members rate their knowledge of AI/ML as basic and did not receive any formal training. The vast majority of participants expressed interest in further AI/ML training, preferring online courses, workshops, and hands-on sessions. Nevertheless, concerns about AI/ML in pediatric surgery were high, especially regarding data bias.

Given the rapid advancements in artificial intelligence (AI) and machine learning (ML) technologies, it is essential for academic surgical societies to proactively incorporate AI/ML education into their core mission [8]. Pediatric surgeons are uniquely positioned to identify which AI-driven innovations can have the most significant impact on patient outcomes. However, without foundational knowledge in AI/ML, they may remain sidelined from these developments, limiting their ability to influence how these technologies evolve in pediatric surgery [1,9]. For the first time, this survey offers valuable insights into the current state of AI/ML knowledge, usage, and attitudes among ESPES members.

Education and training in AI/ML serve three critical purposes. First, they enable surgeons to leverage these technologies to improve surgical precision, decision-making, and, ultimately, patient outcomes. Second, they empower surgeons to actively contribute to the development and refinement of AI/ML tools, ensuring that such tools are designed with the specific challenges of pediatric surgery in mind. Lastly, by taking a proactive stance on AI/ML education, academic societies like the ESPES can ensure their members remain at the forefront of medical innovation, maintaining the highest standards of care and clinical excellence [10].

The present survey of ESPES members explored their readiness and attitudes toward AI/ML technologies in pediatric surgery. While there was widespread optimism about the potential impact of AI/ML, the survey identified significant barriers to adoption.

Knowledge Gaps: A substantial portion of respondents reported limited familiarity with AI/ML technologies, underlining the urgent need for educational interventions. Training programs tailored to pediatric endoscopic surgery could bridge this knowledge gap and empower surgeons to use AI effectively in their clinical practices.

Barriers to Adoption: Consistent with findings from other surgical specialties, the primary barriers to AI adoption in pediatric surgery were the high costs of AI implementation, lack of appropriate infrastructure, and limited access to training. Addressing these barriers requires coordinated efforts at both institutional and organizational levels, with a focus on investment in AI technologies, providing access to AI tools, and establishing a robust training framework [11,12].

Ethical and Legal Considerations: Ethical concerns surrounding AI were also noted, particularly regarding the potential for AI to diminish surgeon autonomy or introduce bias into clinical decision-making. These concerns must be addressed by transparent designs of AI systems, strict regulations, and the integration of ethical training within AI education. Surgeons need to be well versed in the ethical implications of AI use, including issues related to patient privacy, informed consent, and the accountability of AI-driven decisions [13,14].

On a positive note, this survey revealed a strong interest in further AI/ML training, indicating a clear demand for targeted educational programs to address these barriers and capitalize on the enthusiasm for AI integration.

Given these findings, academic societies like the ESPES are uniquely positioned to lead in integrating AI/ML education into their academic offerings. A comprehensive curriculum should cover both theoretical and practical aspects of AI/ML technologies, ensuring that pediatric surgeons not only understand the potential applications, but can also utilize AI tools effectively in their day-to-day clinical practice.

Workshops, seminars, and online courses should introduce the fundamentals of AI and machine learning, with a focus on how these technologies are currently applied in minimally invasive surgery (MIS) [15]. Topics should include AI applications in image recognition, robotic surgery, and predictive analytics for patient outcomes, specifically within the pediatric population.

Practical training is essential for surgeons to gain hands-on experience with AI tools. Simulation-based learning can teach surgeons how to interact with AI-powered devices, as well as interpret and productively integrate AI feedback into live endoscopic procedures [11]. By offering these interactive learning environments, the ESPES can ensure that surgeons are equipped to navigate the complex AI landscape.

Understanding the ethical and legal aspects of AI in surgery is paramount. AI systems must be implemented in ways that protect patient data privacy, uphold consent standards, and ensure that the technology complements, rather than replaces, human clinical judgment. Courses focusing on the ethical and regulatory frameworks surrounding AI can reduce resistance and build confidence in the safe deployment of AI/ML solutions in clinical practice [14].

AI will most likely become an integral part of the future of endoscopic surgery, particularly in the realm of minimally invasive procedures where precision, real-time decision support, and advanced image analysis are critical [16,17]. The participation of surgeons in research and development should therefore be encouraged to facilitate the exploration of future AI applications in pediatric MIS. Involvement in AI research allows surgeons to contribute to developing more refined, efficient tools specifically designed for pediatric patients. Furthermore, engaging with AI research fosters a deeper understanding of how AI solutions are designed, validated, and integrated into clinical workflows, empowering surgeons to critically evaluate and optimize AI tools for their use [15,18].

One limitation of the present study is that only 144 out of 307 ESPES members participated in the survey, and 125 participants answered AI/ML-related questions. Moreover, with respondents recruited from a scientific conference, participants working in academic institutions were overrepresented. Further related research projects should also focus on colleagues working in community hospitals or private practice.

## 5. Conclusions

This survey indicates that the majority of participants only has basic AI/ML knowledge, but is interested in further AI/ML training. Moreover, there are certain concerns mainly regarding ethical issues and the reliability and accuracy of AI/ML systems. We therefore highlight the need for further multi-professional education and training of ESPES members to effectively adopt and utilize AI/ML technologies.

In the future, the ESPES can play a pivotal role by offering a structured curriculum that includes AI workshops, seminars, simulation-based learning, continuous professional development, and research opportunities. By addressing knowledge gaps and overcoming educational barriers, the ESPES can ensure that its members remain at the cutting edge of medical innovation, leading the future of pediatric endoscopic surgery through the intelligent and ethical use of AI. Future research could focus on the effects of structured AI/ML education.

## Data Availability

Raw data supporting the conclusions of this article will be made available by the authors on request due to confidentiality reasons.

## Supplementary Materials

The following supporting information can be downloaded at: https://www.mdpi.com/article/10.3390/children12010006/s1, Figure S1: The influence of age group (a), years of experience in pediatric surgery (b), and primary institution (c) on the general understanding of AI/ML. Figure S2: The influence of age group on concerns towards the implementation of AI/ML.
