# Supplementary figures and images for: Artificial Intelligence (AI) Competency and Educational Needs: Results of an AI Survey of Members of the European Society of Pediatric Endoscopic Surgeons (ESPES)
Source: Children (Basel). 2024 Dec 24;12(1):6. doi: 10.3390/children12010006 (PMC11764318; doi:10.3390/children12010006)

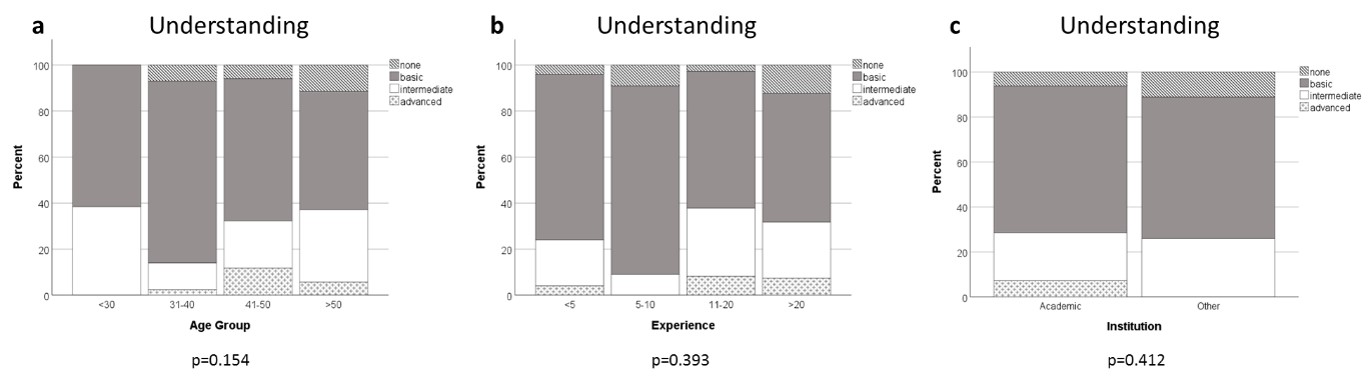

Supplement: Supplementary file 1 [file children-12-00006-s001.zip › Supplementary Figure S1.jpg]

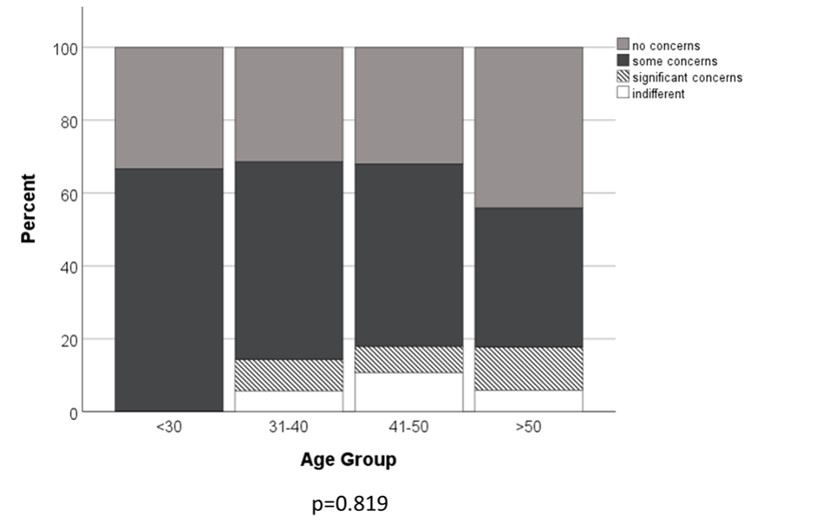

Supplement: Supplementary file 1 [file children-12-00006-s001.zip › Supplementary Figure S2.jpg]
